# Supplementary material for: Association between oral microbiome and seven types of cancers in East Asian population: a two-sample Mendelian randomization analysis
Source: Front Mol Biosci. 2023 Nov 21;10:1327893. doi: 10.3389/fmolb.2023.1327893 (PMC10702768; doi:10.3389/fmolb.2023.1327893)
Supplement: Supplementary file 1 [file DataSheet1.ZIP › Supplementary Materials/MR plots of saliva/saliva═╝/colorectal cancer/pheno.3076_to_colorectal cancer_forest.pdf]

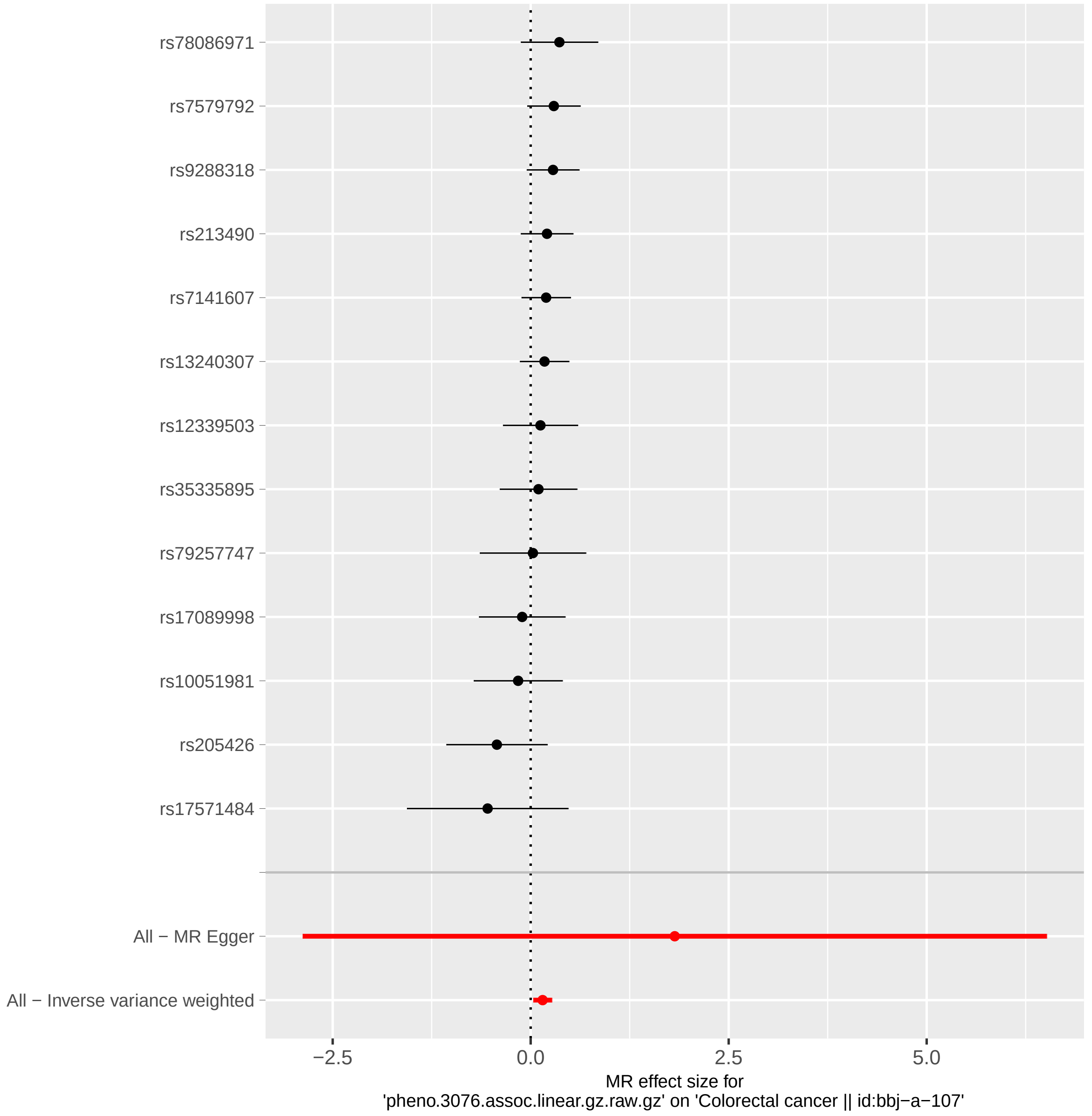

rs78086971
rs7579792
rs9288318
rs213490
rs7141607
rs13240307
rs12339503
rs35335895
rs79257747
rs17089998
rs10051981
rs205426
rs17571484
All − MR Egger
All − Inverse variance weighted
−2.5
0.0
2.5
5.0
MR effect size for
'pheno.3076.assoc.linear.gz.raw.gz' on 'Colorectal cancer || id:bbj−a−107'
